# Conductive GelMA–Collagen–AgNW Blended Hydrogel for Smart Actuator

**DOI:** 10.3390/polym13081217

**Published:** 2021-04-09

**Authors:** Jang Ho Ha, Jae Hyun Lim, Ji Woon Kim, Hyeon-Yeol Cho, Seok Geun Jo, Seung Hyun Lee, Jae Young Eom, Jong Min Lee, Bong Geun Chung

**Affiliations:** 1 Department of Mechanical Engineering, Sogang University, Seoul 04107, Korea; hajangho1991@naver.com; 2 Department of Biomedical Engineering, Sogang University, Seoul 04107, Korea; boy6635@naver.com (J.H.L.); rlawldns68@naver.com (J.W.K.); 3 Department of Bio & Fermentation Convergence Technology, Kookmin University, Seoul 02707, Korea; chohy@kookmin.ac.kr; 4 Division of Chemical Industry, Yeungnam University College, Daegu 42415, Korea; 96tjrrms@naver.com (S.G.J.); jirovsvovan@naver.com (S.H.L.); djawodud40@ync.ac.kr (J.Y.E.)

**Keywords:** conductive blended hydrogel, gelatin methacrylate, collagen, silver nanowire, molecule release

## Abstract

Blended hydrogels play an important role in enhancing the properties (e.g., mechanical properties and conductivity) of hydrogels. In this study, we generated a conductive blended hydrogel, which was achieved by mixing gelatin methacrylate (GelMA) with collagen, and silver nanowire (AgNW). The ratio of GelMA, collagen and AgNW was optimized and was subsequently gelated by ultraviolet light (UV) and heat. The scanning electron microscope (SEM) image of the conductive blended hydrogels showed that collagen and AgNW were present in the GelMA hydrogel. Additionally, rheological analysis indicated that the mechanical properties of the conductive GelMA–collagen–AgNW blended hydrogels improved. Biocompatibility analysis confirmed that the human umbilical vein endothelial cells (HUVECs) encapsulated within the three-dimensional (3D), conductive blended hydrogels were highly viable. Furthermore, we confirmed that the molecule in the conductive blended hydrogel was released by electrical stimuli-mediated structural deformation. Therefore, this conductive GelMA–collagen–AgNW blended hydrogel could be potentially used as a smart actuator for drug delivery applications.

## 1. Introduction

Hydrogels have widely been used in biological applications due to their hydrophilicity and excellent physical–chemical properties [1,2,3]. The structure and characteristics of hydrogels are similar to the three-dimensional (3D) microenvironment of living tissues [4,5,6]. In particular, natural hydrogels (e.g., collagen, hyaluronic acid, and fibrin) made by extracellular matrix (ECM) components have good biocompatibility and biodegradability [7]. Moreover, they have only a few side effects, such as immune and inflammatory reactions during transplantation [8,9,10]. Despite their various advantages, the natural hydrogels still have several limitations, such as weak mechanical properties [11,12], difficulty of control and reproduction due to batch-to-batch variation [13], and expensive production costs [14]. To solve these limitations, the blended hydrogels are fabricated to form a stabilized structure that crosslinks via physicochemical bonds.

Gelatin methacrylate (GelMA), which is made by bonding a methacrylate group and an amine-containing gelatin group, shows excellent mechanical properties [15,16] and biocompatibility [17]. In the previous study, GelMA and chitosan hydrogels were mixed to culture bone mesenchymal stem cells [18]. The use of natural hydrogel-based biomaterial (e.g., chitosan, gelatin) is limited as a scaffold due to its poor mechanical properties. The blended GelMA–chitosan hydrogel improved mechanical properties compared to the gelatin–chitosan hydrogel, and its biocompatibility was demonstrated by analysis of the spread area of bone mesenchymal stem cells on the blended hydrogel surface. Additionally, the blended GelMA–alginate hydrogels were fabricated by an extrusion-based bioprinting method [19]. GelMA was mixed with alginate hydrogel to optimize printing conditions. It demonstrated that the GelMA–alginate blended hydrogel showed higher printing accuracy (over 90%), and the adipose-derived stem cells encapsulated within the GelMA–alginate blended hydrogels were highly viable for 7 days.

The silver nanowire (AgNW), showing excellent conductivity and flexibility, has been widely used as an electrode [20], conductive film [21], and energy harvesting material [22]. The hydrogel containing the AgNW was also employed for flexible skin [23], cell differentiation [24,25], biosensor [26], and actuator [27]. The conductive hydrogel has been previously generated by using collagen and AgNW [28]. It showed that the growth of the cells cultured in collagen with AgNW was largely enhanced, as electric stimulation was applied. Although AgNW is not degradable in vivo, AgNW encapsulated within a 3D hydrogel does not show any toxicity to the cells in vitro. In this study, the conductive blended hydrogel was prepared by mixing GelMA, collagen, and AgNW. The ratio of GelMA, with excellent mechanical property, and collagen, with high biocompatibility, was first optimized and AgNW was subsequently added to improve the electrical conductivity. Rheological analysis demonstrated that the mechanical properties of the conductive blended hydrogels were improved compared to collagen or GelMA–collagen hydrogels. Biocompatibility analysis showed the blood vessel networks of human umbilical vein endothelial cells (HUVECs) encapsulated within the conductive blended hydrogel. Additionally, we confirmed that the molecules inside the conductive blended hydrogel were released by electrical stimuli-mediated deformation of the hydrogel in a controlled manner. Therefore, this conductive GelMA–collagen–AgNW blended hydrogel could be used as a smart actuator to enable control of the molecule or drug.

## 2. Materials and Methods

### 2.1. Preparation of Conductive GelMA–Collagen–AgNW Blended Hydrogel

**Figure 1:**
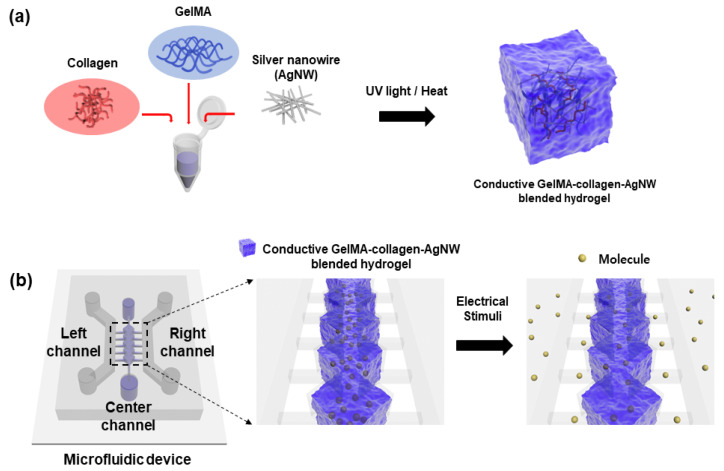
Schematic of the conductive gelatin methacrylate (GelMA)–collagen–silver nanowire (AgNW) blended hydrogel. The protocol for generating the conductive GelMA–collagen–AgNW blended hydrogel (**a**). Electrical stimuli-mediated molecule release from the conductive GelMA–collagen–AgNW blended hydrogel in the microfluidic device (**b**).

To prepare the collagen gel, 98 µL of Dulbecco’s phosphate-buffered saline (DPBS, Thermo Fisher Scientific, Waltham, MA, USA), 80 µL of collagen I rat tail solution (Thermo Fisher Scientific, Waltham, MA, USA), 20 µL of DPBS, and 2 µL of 0.1 M NaOH (Daejung, Shihsing, Korea) were mixed in a vial. A precursor solution was prepared at a temperature of 5 °C to prevent gelation of the collagen. The GelMA (3D materials, Busan, Korea) hydrogel precursor solution was mixed with the collagen gel precursor solution at a constant ratio and then it was uniformly mixed using a vortex (IKA, Staufen, Germany). The desired solution was gelated by irradiating ultraviolet (UV) light for 20 s, using UV exposure (Excelitas, Waltham, MA, USA) for gelation of the GelMA hydrogel precursor solution. In addition, for the gelation of the collagen gel precursor solution when applying heat, the reaction was carried out in a CO_2_ incubator at 37 °C for 30 min. To optimize the ratio of the GelMA–collagen hydrogel, the ratios of GelMA:collagen were changed to 9:1, 7:3, 5:5, 3:7, and 1:9 to confirm the gelation of the hydrogel and the mixing order of gelation. To synthesize the conductive GelMA–collagen–AgNW blended hydrogel (Figure 1a), 10% AgNW solution (Nanophyxis, Gochang, Korea) was added to optimize the ratio of the GelMA–collagen–AgNW hydrogel precursor solution with ratios of 9:3:1, 7:3:1, and 5:5:1 wt%.

### 2.2. Morphological Analysis of the Conductive Blended Hydrogel

To confirm the morphological properties of the conductive blended hydrogel, AgNW, collagen, GelMA hydrogel, GelMA–collagen hydrogel, and GelMA–collagen–AgNW blended hydrogel were observed by using a field emission scanning electron microscope (FE-SEM, JSM-7100F, JEOL, Tokyo, Japan). Each sample was placed on a silicon wafer and dried with a freeze dryer (FDU-1200, Sunil eyela Inc., Seongnam-si, Korea) at −50 °C and the prepared sample was coated with platinum (Pt) for 5 min to suppress the charge-up phenomenon during the measurements. In addition, elemental component analysis of the conductive GelMA–collagen–AgNW blended hydrogel was performed using an energy dispersive X-ray spectrometer (EDS, JSM-7100f, JEOL, Tokyo, Japan).

### 2.3. Rheological Analysis of the Conductive Blended Hydrogel

To evaluate the rheological properties of the conductive GelMA–collagen–AgNW blended hydrogel, 1 mL of collagen, the GelMA hydrogel, GelMA–collagen hydrogel, and GelMA–collagen–AgNW blended hydrogel were gelated in the form of a disk and were then fixed on an 8 mm flat plate. Afterwards, the storage modulus (G’), loss modulus (G”), and composite shear viscosity (η) were analyzed by using a rotational rheometer (TA Instruments Ltd., New Castle, DE, USA). The strain was fixed at 1%, the temperature was set at 25 °C, and the frequency was changed from 0.1 Hz to 10 Hz.

### 2.4. Biocompatibility Analysis of the Conduvtive Blended Hydrogel

To evaluate the biocompatibility of the conductive GelMA–collagen–AgNW blended hydrogel, human umbilical vein endothelial cells (HUVECs) were cultured with endothelial cell growth medium-2 (EGM-2, Lonza, Basle, Switzerland) in a cell culture dish coated with 2% gelatin. The HUVECs were detached by using 0.5% trypsin-ethylenediaminetetraacetic acid (EDTA) (Thermo Fisher Scientific, Waltham, MA, USA) and were then mixed with the precursor solution of the GelMA–collagen–AgNW blended hydrogel. For cell encapsulation, the conductive GelMA–collagen–AgNW blended hydrogel and the HUVEC suspension were injected into the central channel of the microfluidic device (Figure 1b), and UV light was irradiated for gelation. For gelation, the microfluidic device was placed in an incubator for 30 min and then the EGM-2 culture medium was injected into the left and right channel. The medium was changed every day for 5 days. To confirm the viability of the HUVECs that were encapsulated in the GelMA–collagen–AgNW blended hydrogel, the HUVECs were stained for 30 min with a live/dead assay kit and were subsequently observed under a fluorescence microscope. For immunostaining analysis, the HUVECs were cultured for 5 days, washed with DPBS and were then fixed at room temperature for 30 min with 4% paraformaldehyde (CureBio, Seoul, Korea). Afterwards, 1% Triton X-100 (SAMCHUN, Seoul, Korea) was added and the cells were washed with DPBS. To prevent non-specific binding, the cells were treated with 2% bovine serum albumin (BSA, Thermo Fisher Scientific, Waltham, MA, USA) for 2 h. Finally, the cells were stained with Alexa Fluor 594 phalloidin (Thermo Fisher Scientific, Waltham, MA, USA) and 4′,6-diamidino-2-phenylindole (DAPI, Thermo Fisher Scientific, Waltham, MA, USA), and the images of the cells were observed with a confocal microscope.

### 2.5. Statistical Analysis

Statistical analysis was performed using student *t*-test analysis. The differences between the samples were determined as significant (* *p* < 0.05, ** *p* < 0.01). Quantitative data were expressed as the mean ± standard deviation of at least three iterations.

### 2.6. Measurement of Electrical Properties

To analyze the electrical properties of the conductive GelMA–collagen–AgNW blended hydrogel, electrical stimulation was applied to the collagen gels, GelMA hydrogels, and conductive GelMA–collagen–AgNW blended hydrogels by using a voltage amplifier (HSA4011, NF Co., Yokoyama, Japan). For this study, 1 V was applied for 15 min, 30 min, 45 min, and 60 min, and the mass of the hydrogels was measured using an electronic balance before and after electrical stimulation. To observe the selective release of the molecule by electrical stimulation, the conductive blended hydrogel with the fluorescent molecule was injected into the central channel in the microfluidic device (Figure 1b). The diffusion of the fluorescent molecule in the microchannels was observed in response to electrical stimulation. Moreover, the molecule diffusion from the GelMA–collagen–AgNW blended hydrogel was evaluated with fluorescein isothiocyanate (FITC)-dextran (10 kDa, Sigma-Aldrich, St. Louis, MO, USA), a fluorescent molecule on a glass slide with Cr and Au patterned at a voltage of 1 V for 15 min, 30 min, 45 min, and 60 min. The fluorescence images were obtained with a fluorescence microscope (Olympus, Tokyo, Japan) and were then analyzed by using the Image J (National Institute of Health, Bethesda, MD, USA) software.

## 3. Results and Discussion

### 3.1. Optimization of Conductive GelMA–Collagen–AgNW Blended Hydrogel Conditions

A hydrogel was prepared by mixing UV-curable GelMA with collagen. GelMA and collagen could be gelated by a UV light and heat, respectively. Thus, the crosslinking order of UV light and heat needs to be optimized. The ratios of the GelMA hydrogel precursor solution to the collagen precursor solution were changed to 1:9, 3:7, 5:5, 7:3, and 9:1 (Supplementary Material, Figure S1). When the UV light was first irradiated, the GelMA/collagen ratio was gelated from 9:1 to 5:5 in a stable manner (Supplementary Material, Figure S1a). However, when heat was first applied, the GelMA/collagen ratios of 9:1 and 7:3 were only gelated (Supplementary Material, Figure S1b). The UV laser has a wavelength with thermal energy and the thermal energy generated at this wavelength can be applied to the hydrogel for gelation, as previously described [29]. Therefore, the thermal energy simultaneously affects the gelation of collagen when the GelMA hydrogel is gelated by UV light. Thus, we optimized the gelation condition that the GelMA/collagen hydrogel, at a ratio of 5:5, was gelated, in a stable manner when UV light was first irradiated. To improve the electrical conductivity and mechanical strength, AgNW was added to the GelMA–collagen hydrogel and this gelation condition was also optimized. The ratio of AgNW was fixed at 10% v/v for the total volume of the GelMA–collagen hydrogel, as previously described [24,25]. The ratios of GelMA, collagen, and AgNW were mixed in ratios of 5:5:1, 7:3:1, and 9:1:1 (Supplementary Material, Figure S2). Previously, the GelMA–collagen hydrogel was gelated at a ratio of 5:5. However, the hydrogel to which AgNW was added did not gelate at a ratio of 5:5. In contrast, gelation was proceeded from the 7:3 GelMA–collagen hydrogel ratio. Thus, reliable gelation could not be achieved. In the case of a hydrogel with a GelMA:collagen ratio of 9:1, gelation occurred in a stable manner, but a high GelMA ratio might not be suitable for the cell culture and molecule release, as previously described [30]. Therefore, we finally optimized the ratio of the conductive GelMA–collagen–AgNW blended hydrogel to 7:3:1.

### 3.2. Analysis of Conductive GelMA–Collagen–AgNW Blended Hydrogel Properties

**Figure 2:**
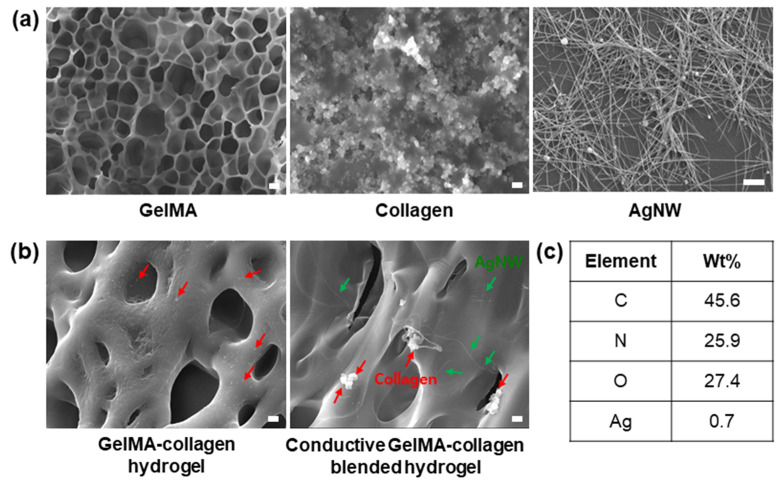
Scanning electron microscope (SEM) images of the conductive GelMA–collagen–AgNW blended hydrogel. SEM images of GelMA, collagen, and AgNW (**a**). SEM images of the conductive GelMA–collagen–AgNW blended hydrogel (**b**). Energy dispersive X-ray spectrometer (EDS) analysis of the conductive GelMA–collagen–AgNW blended hydrogel (Green arrow is AgNW, red arrow is collagen) (**c**). The all scale bars are 1 μm.

To confirm the morphological characteristics of the hydrogel, the cross section of the hydrogel was observed by an FE-SEM (Figure 2). Figure 2a shows the morphologies of GelMA, collagen, and AgNW, respectively. A porous structure was observed in the cross section of the GelMA hydrogel, suggesting that the porous structure could help to transport the molecule in a hydrogel, as previously described [31]. We also observed that collagen was present in the GelMA hydrogel, and the AgNW was randomly distributed in the blended hydrogel (Figure 2b). The AgNW in the blended hydrogel formed a conductive network to improve the electrical properties in the blended hydrogel [20]. In addition, energy dispersive X-ray spectroscopy (EDS) analysis was conducted to analyze the elemental composition of the GelMA–collagen–AgNW blended hydrogel (Figure 2c), showing that 0.7 wt% Ag was contained inside the blended hydrogel.

**Figure 3:**
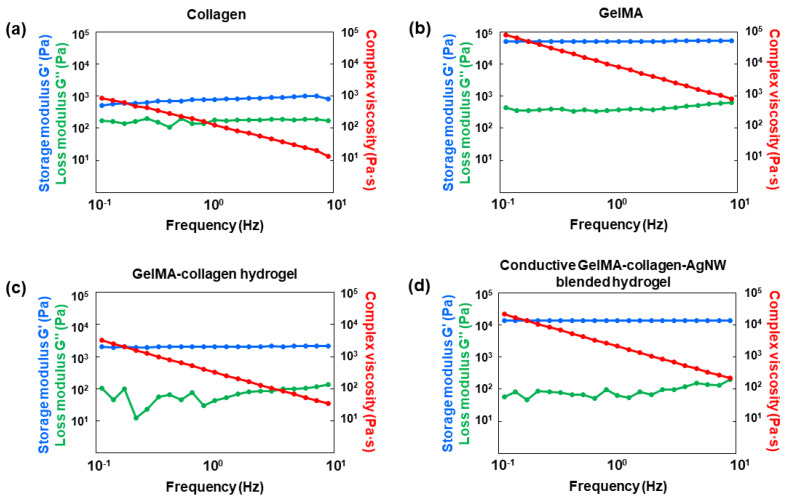
Rheological analysis of the conductive GelMA–collagen–AgNW blended hydrogel. Viscoelastic analysis of the collagen gel (**a**) and the GelMA hydrogel (**b**). Viscoelastic analysis of the GelMA–collagen hydrogel (**c**) and the conductive GelMA–collagen–AgNW blended hydrogel (**d**).

To investigate the rheological characteristics, the conductive GelMA–collagen–AgNW blended hydrogel was analyzed by a rotational rheometer (Figure 3). In comparison to collagen and GelMA, we observed that the storage modulus (G’), loss modulus (G”), and composite shear viscosity (η) of collagen were lower than those of GelMA (Figure 3a,b). The data that the storage modulus (G’) is greater than the loss modulus (G”) indicates that the material almost resembles an elastomer, such as a hydrogel [32]. A high storage modulus (G’) means that the hydrogel has a high crosslink density and strong interfacial bond strength, as previously described [33]. In other words, GelMA has very high viscoelasticity and mechanical strength compared to collagen. In the case of the GelMA–collagen hydrogels, the G’ and G” values were lower than GelMA; however, their mechanical strength was improved in comparison to collagen (Figure 3c). We demonstrated that the mechanical strength of the conductive GelMA–collagen–AgNW blended hydrogel was largely enhanced compared to that of the GelMA–collagen hydrogel (Figure 3d), showing that AgNW increased the mechanical strength of the conductive blended hydrogels [26]. Therefore, we demonstrated that the conductive GelMA–collagen–AgNW blended hydrogel showed excellent mechanical strength.

**Figure 4:**
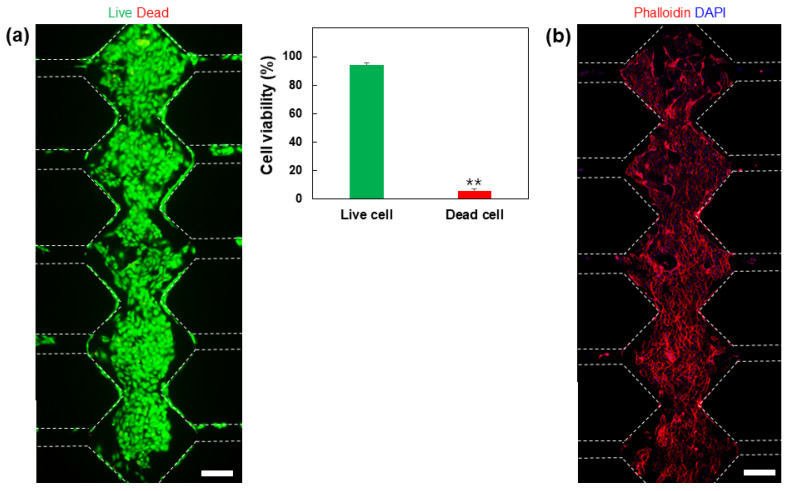
Biocompatibility analysis of the conductive GelMA–collagen–AgNW blended hydrogel. Live (green)/dead (red) fluorescence image of the human umbilical vein endothelial cells (HUVECs) cultured within the conductive GelMA–collagen–AgNW blended hydrogel (** *p* < 0.01) (**a**), and the confocal microscopy image of the HUVECs encapsulated within the conductive GelMA–collagen–AgNW blended hydrogel in the microfluidic device (**b**). The all scale bars are 100 µm.

To confirm the biocompatibility of the conductive blended hydrogel, HUVECs were encapsulated into a 3D GelMA–collagen–AgNW blended hydrogel and were subsequently injected into the microfluidic device (Figure 1b). The cells were cultured for 5 days after gelation in the microfluidic device and the survival of the cells was analyzed by using a live/dead assay kit, which showed that more than 90% of the cells survived (Figure 4a). In addition, to confirm cell growth in the conductive blended hydrogel, HUVECs cultured for 5 days in a microfluidic device were immunostained with phalloidin and 4′,6-diamidino-2-phenylindole (DAPI) (Figure 4b). The immunostaining result showed that HUVECs encapsulated within a 3D conductive blended hydrogel were well grown to form blood vessel networks, suggesting that the GelMA–collagen–AgNW blended hydrogel showed excellent biocompatibility. AgNW conductivity could be increased as an aligned form [34,35]. However, our conductive blended hydrogels were physically mixed to encapsulate the cells in a 3D microenvironment. Since the physical mixing is proceeded by pipetting, AgNW is randomly distributed in a 3D conductive blended hydrogel. In the previous study, it was conducted to mix AgNWs and reduced graphene oxide (rGO) to generate a blended hydrogel for increasing the electrical conductivity [24]. Although rGO and AgNWs were randomly distributed in a 3D hydrogel that was similar to our study, the neural stem cells were successfully differentiated into nerve cells through electrical stimulation.

### 3.3. Conductive Smart Actuator of GelMA–Collagen–AgNW Hydrogel

**Figure 5:**
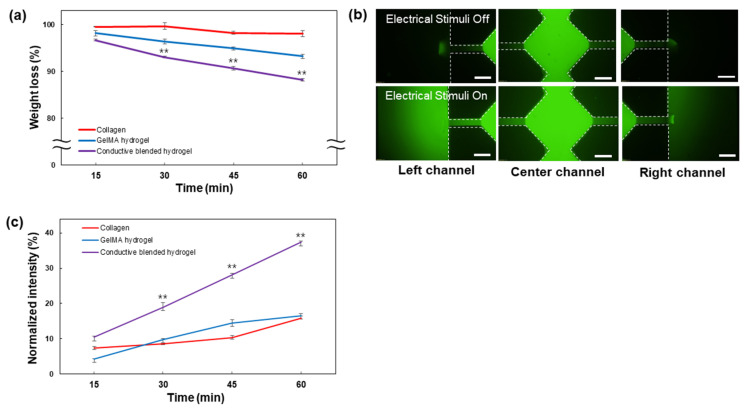
Analysis of the molecule release of the conductive GelMA–collagen–AgNW blended hydrogels. The weight loss analysis of the GelMA–collagen–AgNW blended hydrogel in response to an electrical stimulation (** *p* < 0.01) (**a**), the diffusion images of the fluorescent molecule in the microfluidic device with an electrical stimulation (**b**), and the diffusion rate of the fluorescent molecule every 15 min with electrical stimulation (** *p* < 0.01) (**c**). The all scale bars are 300 µm.

The mass change of the conductive GelMA–collagen–AgNW blended hydrogel in response to electrical stimulation time was analyzed (Figure 5). The mass change of the collagen and GelMA hydrogel was negligible for 60 min, when 1 V was applied. In contrast, the mass of the conductive GelMA–collagen–AgNW blended hydrogel was decreased every 15 min (Figure 5a). When a voltage was applied to the conductive blended hydrogel, the ions could be moved within the blended hydrogel, showing an osmotic pressure between the outside and inside of the blended hydrogel. It resulted in a decrease in the mass, since the water contained in the hydrogel escaped, as previously described [36]. Thus, the conductive GelMA–collagen–AgNW blended hydrogel enabled the control of the molecule release through the contraction and expansion process of the blended hydrogel. The electric current applied through the blended hydrogel does not exceed a 1 mA current which is barely perceptible in people [37]. Therefore, the voltage and current we used in this study are safe for humans. The fluorescent molecule of the blended hydrogel in the microfluidic device did not diffuse to both microchannels when an electric field was not applied (Figure 5b). In contrast, when an electric field was applied, the fluorescent molecule was diffused into both microchannels. We finally demonstrated that the molecule release within the conductive GelMA–collagen–AgNW blended hydrogel was increased by electrical stimulation (Figure 5c). When a voltage is applied to a conductive blended hydrogel, the blended hydrogel contracts and expands, acting as an electrical stimuli-mediated actuator to release the molecule in a controlled manner. The polymer, such as a shape memory polymer (SMP), can swell or recover its original shape through interaction with solvents, as previously described [38,39]. In contrast, our conductive GelMA–collagen–AgNW blended hydrogel is used in a cell culture medium solution without any toxic solvent interaction to release the molecules through contraction and expansion by electrical stimulation (Figure 5). In our previous study, we confirmed that the movement of nanoparticles in a 3D hydrogel containing collagen and AgNW was not observed until electrical stimulation was applied [27]. However, when electrical stimulation was applied, nanoparticles in the hydrogel were released to target specific cancer cells. Although SMP may be a useful actuating material, we used a conductive GelMA–collagen–AgNW blended hydrogel, without any toxic solvent interaction, for 3D cell encapsulation and electrical stimuli-mediated molecule release applications.

## 4. Conclusions

We developed a conductive GelMA–collagen–AgNW blended hydrogel with excellent mechanical strength and biocompatibility. The rheological analysis demonstrated that the storage modulus and loss modulus of the GelMA–collagen hydrogel were significantly improved to 13.77 kPa and 201.8 kPa, respectively, compared to collagen. Additionally, the addition of a AgNW increased the storage modulus from 2.2 kPa to 13.77 kPa, suggesting that AgNW could play an important role in improving the electrical conductivity and the mechanical strength of the blended hydrogel. We also observed the vascular network of HUVECs encapsulated within 3D conductive blended hydrogels in the microfluidic device. Additionally, we demonstrated that the fluorescent molecules in the conductive blend hydrogel were selectively released by electrical stimulation, suggesting that the conductive GelMA–collagen–AgNW blended hydrogel could act as an actuator to regulate the release of molecules in a controlled manner.

## Data Availability

The data presented in this study are available on request from the corresponding author.

## Supplementary Materials

The following are available online at https://www.mdpi.com/article/10.3390/polym13081217/s1, Figure S1: analysis of the gelation of the hydrogel with respect to the GelMA/collagen ratio. Figure S2: analysis of the gelation of the hydrogel with respect to the GelMA/collagen/AgNW ratio.
