# Supplementary material for: Conductive GelMA–Collagen–AgNW Blended Hydrogel for Smart Actuator
Source: Polymers (Basel). 2021 Apr 9;13(8):1217. doi: 10.3390/polym13081217 (PMC8068890; doi:10.3390/polym13081217)
Supplement: Supplementary file 1 [file polymers-13-01217-s001.pdf]

polymers

MDPI

Supporting Information

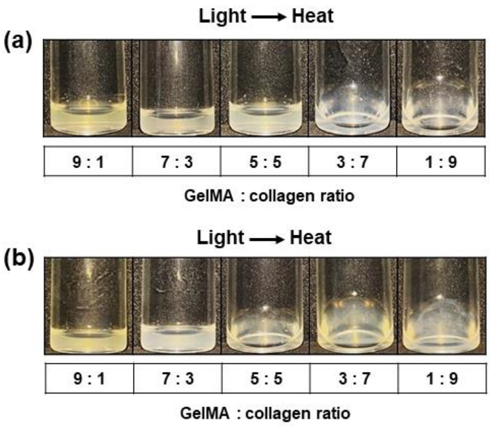

(c)

GelMA : Collagen ratio	Process	Gelation
9 : 1	Light → Heat	O
	Heat → Light	O
7 : 3	Light → Heat	O
	Heat → Light	O
5 : 5	Light → Heat	O
	Heat → Light	X
3 : 7	Light → Heat	X
	Heat → Light	X
1 : 9	Light → Heat	X
	Heat → Light	X

Figure S1. Analysis of the gelation of the hydrogel with respect to the GelMA/collagen ratio. Images of gelation of the hydrogel by applying an UV light after the heat (**a**). Images of the gelation of the hydrogel by applying heat after UV light (**b**). Table of the gelation of the hydrogel (**c**).

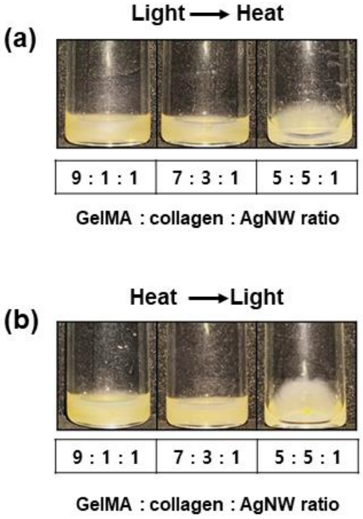

(c)

GelMA : Collagen : AgNW ratio	Process	Gelation
9 : 1 : 1	Light → Heat	O
	Heat → Light	O
7 : 3 : 1	Light → Heat	O
	Heat → Light	O
5 : 5 : 1	Light → Heat	X
	Heat → Light	X

Figure S2. Analysis of the gelation of the hydrogel with respect to the GelMA/collagen/AgNW ratio. Images of the gelation of the hydrogel by applying an UV light after the heat (**a**). Images of the gelation of the hydrogel by applying heat after the UV light (**b**). Table of the gelation of the hydrogel (**c**).
